# Application of Creep Feed and Phytase Super-Dosing as Tools to Support Digestive Adaption and Feed Efficiency in Piglets at Weaning

**DOI:** 10.3390/ani11072080

**Published:** 2021-07-12

**Authors:** Sophie A. Lee, Erica Febery, Pete Wilcock, Michael R. Bedford

**Affiliations:** 1 AB Vista, Marlborough, Wiltshire SN8 4AN, UK; Pete.Wilcock@abvista.com (P.W.); Mike.Bedford@abvista.com (M.R.B.); 2 Drayton Animal Health, Stratford-Upon-Avon, Warwickshire CV37 9RQ, UK; Erica.Febery@draytonah.co.uk

**Keywords:** phytase, creep feed, pH, cortisol, *myo*-inositol

## Abstract

**Simple Summary:**

Weaning is a highly stressful period in the pig production cycle, often resulting in digestive dysfunction, reduced performance and economic losses. Feeding strategies pre- and post-weaning can be used to modulate gut development and function, thereby reducing the risk of gastrointestinal disorders. The current study investigated whether offering creep feed to the suckling piglet or supplementing high levels of phytase post-weaning could reduce stress and support piglet adaption to weaning. Results suggest that while these approaches did not directly reduce stress, they can improve feed efficiency in weaning piglets by enhancing gastric function, phytate breakdown and *myo*-inositol provision. Therefore, application of these practices may allow piglets to better adapt to weaning and promote performance thereafter.

**Abstract:**

A total of 64 piglets were used in a 35-day study to evaluate whether creep feeding piglets on the sow or super-dosing phytase to piglets post-weaning can be used as a tool to reduce stress and support adaption to weaning. Treatments consisted of creep or no creep feed being offered pre-weaning and with or without phytase supplementation at 2000 FTU/kg post-weaning. Blood samples were collected from eight piglets per treatment on days 0 (weaning), 7 and 21 post-weaning to determine plasma cortisol and *myo*-inositol concentrations. Four piglets per treatment (*n* = 16) were administered Heidelberg pH capsules 1 week prior to weaning, on the day of weaning, as well as 7 days and 21 days post-weaning, with readings monitored over a 3 h period. In the first week post-weaning, creep-fed piglets had higher daily gains (0.23 vs. 0.14 kg/d, *p* < 0.05) and a lower feed conversion ratio (FCR, 0.99 vs. 1.35, *p* < 0.01), compared to non-creep-fed pigs. At 21 days post-weaning, irrespective of creep feed, phytase supplementation reduced FCR (1.10 vs. 1.18, *p* = 0.05) of piglets. Average real-time stomach pH was lower in creep-fed piglets at 1 week prior to weaning (pH 3.2 vs. 4.6, *p* < 0.001) and on day of weaning (pH 3.1 vs. 3.7, *p* < 0.01). Following weaning, phytase reduced average stomach pH of piglets at days 7 (pH 2.6 vs. 3.3, *p* < 0.001) and 21 (pH 2.2 vs. 2.6, *p* < 0.01). Both cortisol and *myo*-inositol concentrations in plasma decreased with age; however, cortisol levels were unaffected by either treatment. Plasma *myo*-inositol concentrations were higher in creep-fed piglets at day of weaning (*p* < 0.05) and with phytase super-dosing on day 21 (*p* < 0.001). These findings demonstrate that both creep feeding and phytase super-dosing are useful practices to encourage better adaption to weaning and support piglet performance. This response was not related to reduced stress in piglets, as determined by cortisol levels, but instead appears to relate to improved gastric conditions for digestion, phytate degradation and *myo*-inositol provision in piglets.

## 1. Introduction

Weaning exposes piglets to numerous stressors when they are handled, transported and placed in a new social environment and are transitioning from milk to solid feed. In most commercial farms, weaning is not a gradual process but one that takes immediate effect, generally at 3 to 4 weeks of age. Following these abrupt changes, the period following weaning is characterised by a temporary decline in feed intake and a growth check [1]. During this time, piglets have an increased susceptibility to gut disturbances, infections and diarrhoea [2]. Traditionally, in-feed antibiotics as growth promoters has been the primary approach to overcome the challenges of weaning. However, in Europe, the use of antibiotics at sub-therapeutic growth promoting levels has been banned (Regulation (EC) No. 1831/2003), and authorities worldwide are looking to limit further the therapeutic use [3,4] of antibiotics. Accordingly, great efforts have been made to find alternatives, such as using feeding strategies to mitigate gastrointestinal disorders and improve productive parameters [5,6].

Replacing milk with solid feed, piglets must adapt to more complex and less digestible plant-based diets, comprising variable amounts of antinutritional factors, such as phytate. The undesirable effect of phytate on the availability of phosphorus (P) and other nutrients has been well-documented and reviewed [7,8,9], with data showing that an increase of dietary phytate post-weaning [10] has a negative impact on piglet performance. Phytate-degrading enzymes, such as phytases, are routinely added to pig diets to degrade phytate, increasing availability and absorption of P and reducing its secretion into the environment [11]. Typically phytases have been added to diets at a standard rate of 500 FTU/kg to target a moderate release of available P [12]. However, it has been demonstrated that using super-doses of phytase, at 3 to 5 times higher than the standard phytase dose, can lead to growth responses beyond what would be expected from the additional release of P [13,14,15]. A study in broilers suggested that 30 to 35% of the super-dosing response could be attributed to the generation of *myo*-inositol from the complete dephosphorylation of phytate [16]. In mammals, *myo*-inositol plays an important role in several metabolic and regulatory processes, including lipid and glucose metabolism, which may promote a growth response in some animals (for review see Gonzalez-Uarquin et al. [17] and Lee and Bedford [18]). Lipid-bound inositol, phosphatidylinositol, is an important component of cell membranes and plays a critical role in maintaining epithelial cell integrity and function in the gastrointestinal tract, particularly during times of challenge. Accordingly, a recent study by Moran et al. [19] suggested that *myo*-inositol might be a conditionally essential nutrient for young pigs during the weaning process.

Creep feeding during the suckling period is also considered a useful management tool to aid the transition of piglets from sow’s milk to solid feed and, thus, to reduce the identified post-weaning lag. In suckling piglets, acidification of stomach contents is largely due to the presence of lactic acid, resulting from lactose fermentation, which may in part suppress hydrochloric acid (HCl) secretion [20]. Piglets with access to creep feed while suckling have heavier stomachs and greater HCl and pepsin production capacity than non-creep-fed pigs [21], suggesting diet-induced development of the gastrointestinal tract of piglets. This should enable piglets to adapt to weaning more readily and improve early post-weaning feed intake and weight gain; however, results have been shown to be inconsistent [22,23,24,25]. Although changes to stomach function have been less studied in comparison to the intestine at weaning, gastric efficiency is crucial for overall digestive success. In a previous study using real-time pH capsules [26], pigs fed diets supplemented with 2500 FTU/kg phytase were able to maintain stomach pH through a potentially challenging period associated diet phase change. Therefore, it is possible that both creep and phytase could regulate stomach pH during weaning, and as a result, support feed efficiency in the young pig. Therefore, the objective of this study was to evaluate the use of creep feed and phytase super-dosing as tools to regulate gastric pH and improve post-weaning performance, thereby alleviating stress responses and supporting piglet adaption to weaning.

## 2. Materials and Methods

### 2.1. Experimental Diets and Treatments

Sixty-four piglets were allocated to one of four treatment groups in a 2 × 2 factorial arrangement consisting of creep or no creep feed being offered pre-weaning and post-weaning diets with or without phytase supplementation. In treatments where a commercial creep feed was offered, piglets were provided ad libitum access via a feeder from 2 weeks of age. Composition of creep feed was based on wheat, soya, fishmeal and lactose, and was analysed to contain 20.8% crude protein and 16.2 MJ/kg DE. Creep feed also comprised a digestibility enhancer, which included an *E. coli* phytase at 100 FTU/kg, an endo-1,4-β-xylanase at 70 AXC/kg and an endo-1,3(4)-β-glucanase at 100 AGL/kg.

Post-weaning pigs were fed pelleted starter diets (Table 1), without or with phytase (0 or 2000 FTU/kg). The phytase used in this experiment was an enhanced *E. coli* phytase (Quantum Blue; AB Vista, Marlborough, UK), with an expected activity of 5000 FTU/g. In-feed phytase activities were analysed by ELISA (performed by AB Vista Lab Services), and met the expected minimum at 2580 FTU/kg for phytase supplemented diets. The non-supplemented control diet had an analysed phytase activity of 159 FTU/kg, suggesting that there may have been some cross-contamination in the feed. Starter diets and water were provided ad libitum from days 0 to 21 of the study.

### 2.2. Animals and Housing

Eight litters from a mix of Landroc (Red Duroc × Landrace) and pure Landrace sows were selected based on a standardised litter age to be used in this study. Piglets were housed in farrowing crates with their litters at Countess Wells Farm, UK until weaning at approximately 28 days of age. Two weeks before weaning, four of the selected litters were offered a commercial creep feed and the other four litters were not offered any creep feed. At day of weaning (d0), eight piglets per litter (64 in total) were selected based on an equal sex ratio, transported to Drayton Animal Health and housed in one room with 16 pens, and four piglets per pen (including one pH capsuled piglet). Male and female piglets were penned by sex. Eight pens were allocated to piglets that were previously creep-fed, and the other eight pens were assigned to piglets that were not offered any creep feed. Starter diets with and without phytase were evenly split between pens that contained piglets previously fed or not fed creep (four pens per treatment). Pigs were exposed to a 16 h light regime and an initial housing temperature of 26 °C, which decreased every seven days by 1.5 °C until the end of study on day 21. All pigs and feed were weighed on days 0 (prior to feeding starter diets), 7 and 21 to calculate the average daily gain (ADG), average daily feed intake (ADFI) and feed conversion ratio (FCR, ADFI/ADG), respectively. In addition, on day 0, feed intake per pen was recorded every 3 to 4 h over a 24 h period to determine piglet willingness to eat.

### 2.3. Capsule Administration

The Heidelberg pH Diagnostic System (fifth generation) from Heidelberg Medical, consisting of a pH capsule and transceiver, was used to capture real-time pH readings. One week prior to weaning, 16 piglets (eight creep-fed piglets and eight non-creep-fed piglets) were selected based on an equal sex ratio for capsule administration, with eight pigs being dosed either in the morning (AM) or afternoon (PM). Following weaning and transfer of piglets to Drayton Animal Health on day 0, the same piglets were administered capsules again at their respective AM/PM times. Similarly, on days 7 and 21, one pig per pen which had previously received capsules was administered capsules again. On days 0 and 7, only seven Heidelberg medallions were able to be calibrated and used over the AM and PM capsuling sessions. This resulted in obtaining pH data from 14 out of 16 selected piglets. On day 21, 16 piglets were pH capsuled over three batches to enable all pigs to be capsuled.

Pigs were orally dosed using a bespoke bolus gun and pH readings were monitored over a 3 h period. During pH monitoring, the transceiver was kept in close proximity to the pig to allow data capture. During this time, pigs were individually housed so to prevent damage to the transponder. Pigs were still able to see one another and had ad libitum access to feed and water. Capsule readings were collected every second and aggregated into 5 min averages prior to analysis. Readings of pH 0 were removed from the dataset, as these were not considered ‘true’ values. Anomalies, determined by values residing outside 3 × the root mean square error (RMSE), were also removed from the dataset prior to statistical analysis.

### 2.4. Blood Sampling

Blood samples were taken from all 16 piglets designated for pH assessment, as well as one additional pig per pen on days 0, 7 and 21. Blood sampling of the capsuled piglets took place during pH monitoring. A target of 10 mL of blood was collected from the jugular vein into lithium heparin vacutainers. Tubes were carefully inverted to ensure full mixing of the sample and prevent clotting, and immediately placed on ice until processing. Erythrocytes were pelleted by centrifugation at 1500× *g* for 10 min and an aliquot washed by mixing with two volumes of ice-cold perchloric acid, iced for approximately 15 min, followed by centrifugation at 12,000× *g* for 10 min. Two aliquots of plasma collected from each tube. The first aliquot was processed by Nottingham Trent University for measurement of cortisol by ELISA (Enzo Life Sciences Inc., New York, NY, USA; ADI-900-071). The second aliquot was processed by the University of East Anglia for determination of *myo*-inositol concentration by HPLC pulsed amperometry (HPLC-PAD), as described previously [27].

### 2.5. Statistical Analysis

Data were analysed by ANOVA using the fit model platform of JMP Pro 15.1 (SAS Institute Inc., Cary, NC, USA). Pen represented the experimental unit for performance data, while pig was the experimental unit for pH and blood parameters. The statistical model included creep feed and phytase as fixed effects. For performance data, the interaction between creep feed and phytase was also included in the model and pen as a random effect. The effect of creep feed on post-weaning diet intake over the first 24 h was analysed using creep and time as fixed effects. Treatment means were separated using student’s t-test, with significance accepted at *p* ≤ 0.05. The main effects are presented as least squares means (LSM) with their pooled standard errors of the mean (SEM).

## 3. Results

### 3.1. Performance

**Figure 1:**
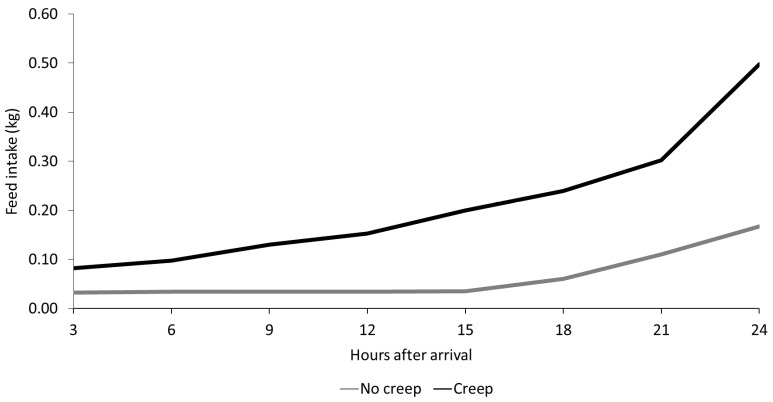
Effect of creep feed on post-weaning cumulative intake of starter diets over 24 h from arrival on site. Data represent means of eight replicate pens per treatment group (creep and no creep).

During the study, there were no pig mortalities, and therefore, survival was 100% for all treatments. Considering feed intake over the first 24 h post-weaning and arrival on site (Figure 1), piglets previously fed creep consumed starter diets more readily than piglets not fed creep (*p* < 0.001). However, for both groups, intake started to increase considerably from 21 h post-weaning (*p* < 0.001) and after the first week ADFI was comparable across treatments (Table 2). However, at day 7 post-weaning, pigs that had previously been creep-fed gained more weight (0.23 vs. 0.14 kg/d, *p* < 0.05) compared to non-creep-fed pigs in this period. Consequently, FCR of pigs fed creep feed prior to weaning was reduced by approximately 27% (0.99 vs. 1.35, *p* < 0.01), compared to non-creep-fed pigs. Phytase supplementation had no significant effect on piglet performance in the first week post-weaning.

Over the 21 days post-weaning, feed intake was not significantly affected by creep feeding prior to weaning or phytase supplementation. Though numerically pigs fed creep or phytase gained 0.85 kg and 0.61 kg more weight, respectively, than non-creep or phytase-fed pigs over the 21 days post-weaning, ADG was not statistically significant between treatments. Nonetheless, supplementing 2000 FTU/kg phytase to diets reduced FCR of pigs by around 8 points (1.10 vs. 1.18, *p* = 0.05), compared to non-supplemented diets. Creep feeding had no effect on overall performance over the 21 days post-weaning.

### 3.2. Real-Time Gastric pH

**Figure 2:**
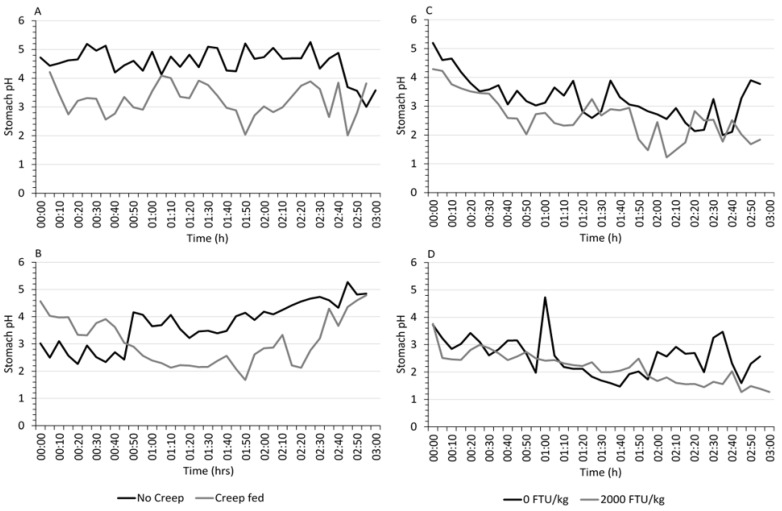
Effect of creep feed and phytase super-dosing on real-time stomach pH of piglets. Data show the average stomach pH readings taken over 3 h from eight pigs per treatment (creep/no creep) at 7 days pre-weaning (**A**), from seven pigs per treatment (creep/no creep) at day of weaning (**B**), from six (0 FTU/kg phytase) and eight (2000 FTU/kg phytase) pigs per treatment at day 7 (**C**) and eight pigs per treatment (0 or 2000 FTU/kg) at day 21 (**D**) post-weaning.

The effect of creep feed at 1 week pre-weaning (Figure 2A) and at day of weaning (Figure 2B), as well as phytase supplementation at 7 days (Figure 2C) and 21 days (Figure 2D) post-weaning on pH capsule readings over the 3 h monitoring period was assessed. During the suckling period, piglets that had been offered creep feed had lower average stomach pH (*p* < 0.01) than those that had no creep (Table 3) at 1 week pre-weaning (pH 3.2 vs. 4.6) and on day of weaning (pH 3.1 vs. 3.7). Following weaning (Table 4), supplementing phytase to diets reduced (*p* < 0.001) average stomach pH of piglets at days 7 (pH 2.6 vs. 3.3) and 21 (pH 2.2 vs. 2.6). Over the course of the study, average gastric pH of sampled pigs declined (*p* < 0.001) by approximately 0.5 pH units from pre-weaning (pH 3.9) to days 0 (pH 3.4), 7 (pH 2.9) and 21 (pH 2.3) post-weaning.

### 3.3. Plasma myo-Inositol and Cortisol

Plasma was collected from pigs at days 0, 7 and 21 post-weaning to measure the concentration of *myo*-inositol and cortisol (Table 5). Pigs in the creep-fed group had approximately 20% higher (*p* < 0.05) plasma *myo*-inositol levels at day of weaning compared to the non-creep feed group. Nonetheless, plasma *myo*-inositol levels drop by around 56% from day of weaning to day 7 post-weaning. Previous feeding of creep had no effect on plasma *myo*-inositol at days 7 and 21. Feeding phytase significantly increased (*p* < 0.001) plasma *myo*-inositol in pigs at day 21, with levels approximately doubling compared to non-phytase-fed pigs. Plasma cortisol levels were around 3.5 and 8 times higher at weaning than at days 7 and 21, respectively. However, creep and phytase treatment had no significant effect on cortisol levels in pigs at any age.

## 4. Discussion

Weaning is arguably the most stressful and challenging period in the production cycle for pigs. Weaning weight is considered an important determinant of post-weaning performance and the time required to reach market weight [28]. Creep feeding is a popular strategy used to bridge the gap between the increasing nutrient demands of the suckling pig and the nutrients supplied by the lactating sow. Despite intake of creep being highly variable among individuals [24,29], it has been shown to stimulate early post-weaning feed intake and weight gain, suggesting that creep might familiarise piglets with solid feed and allow the digestive tract to gradually adapt to a change in feed form and composition. However, studies have shown inconsistent results to creep feed [22,29,30,31,32]. In the current study, weaning weights of piglets not fed creep were on average 500 g lighter than piglets that had been offered creep from day 14. However, this weight difference was not statistically significant and, therefore, does not support the creep feeding effect on weaning weights. However, there was a clear difference between pigs that had been creep-fed in terms of willingness to consume starter diets over the first 24 h post-arrival. Despite there being no statistically significant effect of creep feed on ADFI over the first week, feed efficiency and ADG were enhanced by approximately 27% and 62%, respectively, with creep feeding. This could be attributed to a more suitably adapted digestive tract, enabling piglets to better meet nutritional demands. In addition, it is expected that this would reduce the flow of undigested nutrients to the large intestine, where the undigested protein fraction in particular could be harmful if fermented [33,34]. When piglets consumed creep feed during lactation, Kuller et al. [35] reported higher net absorption capacity in the small intestine 4 days post-weaning, compared to non-eaters. However, Muns and Magowan [36] found no effect of creep intake on gut structure at 1 or 3 week post-weaning. In the present study, the observation of an FCR <1.0 in creep-fed piglets may have been the result of greater water intake, which is known to be highly and positively correlated to feed intake [37].

During suckling, piglets had an average gastric pH of around 4, which is close to the optimum activity for chymosin [38], an important enzyme for milk-clotting. Lactic acid produced from the fermentation of lactose is primarily responsible for the acidification of the stomach of suckling pigs [20], acting as an HCl-production inhibitor until access to the sow is denied and solid feed becomes the main source of nourishment. On the day of weaning, stomach pH dropped to about pH 3.4, which may have been due to the increased stress of piglets as well as the lack of food to buffer the stomach. In both life stages, results of the real-time stomach pH measurements showed that creep feeding significantly reduced gastric pH 7 days pre-weaning (3.24 vs. 4.55) and on the day of weaning (3.08 vs. 3.70), suggesting that HCl secretions were more efficient in these piglets. Cranwell et al. [20] demonstrated in cannulated piglets that with the consumption of creep feed, there was an acid-secretory response and a drop in gastric pH. However, with subsequent intake of milk, this response was reverted. In a recent study, Choudhury et al. [39] found no effect of creep feeding on the pH of digesta collected from the stomach of piglets prior to weaning. Isolation of digesta from the tract prior to taking pH measurements has been shown to interfere with pH determinations [40] due to a change in environmental conditions. In addition, pH measurements taken in situ have shown higher variability and lack of treatment effect compared to real-time measurements in the live animal [26], suggesting that point-in-time measurements are not representative of gastric pH fluidity. With age and an increase in solid feed, chymosin is gradually substituted by pepsin [41], and as such, a lowering of stomach pH by HCl secretions is necessary to achieve optimal digestive function. Therefore, piglets fed creep were more likely to be better prepared for gastric digestion of more complex and less digestible plant proteins once weaned. This was supported by performance data for the first week post-weaning, with creep-fed pigs gaining more weight whilst having similar feed intake.

Cortisol is the main glucocorticoid produced by pigs in response to hypothalamic-pituitary-adrenal (HPA) axis activation [42]. Accordingly, measuring circulating concentrations of cortisol is a standard approach used to evaluate stress and welfare of pigs [43]. The average concentration of cortisol in pigs decreases with age, reaching an asymptote at around 20 weeks of age [44]. In the current study, plasma cortisol levels reduced by approximately 71% from day of weaning to day 7 postweaning, and a further 17% to day 21. Similar to the findings of van der Meulen et al. [45], creep feed did not affect cortisol levels at day of weaning. In addition, phytase treatment showed no effect on cortisol levels, which would have been evident from day 7 post-weaning. Moeser et al. [46] reported that after 24 h, post-weaning serum cortisol levels increased by 95% compared to levels in un-weaned pigs. However, at weaning, these pigs had similar cortisol levels to un-weaned pigs and after 7 days, post-weaning cortisol levels fell back to the control. These results suggest that there may be a time-lag between stress-induced activation of the HPA and the respective rise in circulating cortisol levels. Therefore, this suggests that any potential difference in cortisol production between treatments may have been missed due to the timing of measurements taken.

The supplementation of exogenous phytase to weaned pig diets to reduce the antinutritional effects of phytate, enhance nutrient digestibility and increase growth performance has been well studied [47,48,49,50]. In a number of these studies, the phytase efficacy has been evaluated in response to feeding P-deficient diets, to determine P equivalencies with phytase [11]. Phytase at doses that exceed the standard level is considered to have extra-phosphoric effects due to growth performance improvements that go beyond what can be accounted for by the extra P released [51,52]. Part of this response has been attributed to the destruction of antinutritive phytate esters and the release of *myo*-inositol. Supporting this theory, a number of studies have reported improved feed efficacy in chickens [16,53,54] and pigs [19] with dietary *myo*-inositol supplementation. These performance responses were matched or exceeded with appropriate phytase supplementation. In addition, recent studies have reported a dose-dependent increase in phytate disappearance and *myo*-inositol concentrations in intestinal digesta of piglets with phytase [14,15,55,56]. Free *myo*-inositol is absorbed and can be detected in both portal and peripheral blood of pigs [15,52,56,57].

Plasma *myo*-inositol concentrations were highest at day of weaning and had reduced by more than half the concentration by day 7 post-weaning. It is likely that sow milk had provided some *myo*-inositol to piglets [58]; however, plasma *myo*-inositol concentrations were 20% higher at weaning in piglets fed creep. The commercial creep feed comprised a digestibility enhancer that included an *E. coli* phytase at 100 FTU/kg. However, at such low inclusion rates, it is unlikely that phytase contributed wholly to the provision of the additional *myo*-inositol to piglets. According to Boge and Brækkan [59], *myo*-inositol comprises around 700–800 mg/kg dry weight of fishmeal, depending on the fish species. Therefore, fishmeal included in the creep may have also contributed to the elevated blood *myo*-inositol levels. The positive effect of supplementing *myo*-inositol on feed efficiency during the first 10 days post-weaning has been shown previously [19], suggesting that *myo*-inositol may have a greater metabolic impact in piglets immediately after weaning. Although there was no treatment effect at day 7, plasma *myo*-inositol concentrations were twice as high in piglets fed exogenous phytase at day 21 compared to piglets fed non-supplemented diets. Supporting previous work, these findings indicate that high doses of phytase enable more complete phytate destruction, thereby reducing the anti-nutritive effect of phytate and releasing *myo*-inositol. Moreover, this super-dosing effect translated over to increased feed efficiency in piglets at day 21.

At days 7 and 21 post-weaning, piglets in the current study had an average real-time stomach pH of 2.9 and 2.3, respectively. Nonetheless, stomach pH fluctuated considerably from as low as pH 1.2 to as high as pH 5.2. In a previous study [26], average stomach pH, as measured by real-time pH capsules, was around pH 1.8 in piglets on days 18 and 19 post-weaning, and was unaffected by phytase dosed at 2500 FTU/kg. Variations in pH fluctuations were also less extreme, ranging from around pH 1.0 to 3.6. However, after a diet phase change on day 22, piglets fed phytase were able to maintain average stomach pH at around 1.8, while stomach pH of non-supplemented piglets increased to 2.8 in response to a change in potential buffering capacity of the diet. In the current study, supplementing phytase at 2000 FTU/kg reduced average real-time stomach pH of piglets at days 7 and 21. Encouraging pH to fall more rapidly in the young pig is beneficial to stimulate pepsin activation and protein digestion, thereby limiting the amount of undigested material passing through the tract that can be used as a substrate for putrefactive fermentation [60]. In addition, gastric acidity reduces the number of viable pathogenic bacteria that reach the small intestine [61], and as such, it is an essential first defence mechanism in the animal.

## 5. Conclusions

In summary, no interaction was found between creep feeding and phytase supplementation under the conditions of the study. However, respective practices have encouraged better adaptation to weaning and enhanced feed efficiency at different stages of the piglet production cycle. Although this response was not associated with reduced stress in piglets, as determined by circulating cortisol levels, it may relate to improved gastric function, phytate degradation and *myo*-inositol provision in piglets.

## Figures and Tables

**Table 1:** Ingredient and nutrient composition of starter diets (as is) ^1^.

Ingredient, g/kg	
Barley	100
Wheat whole meal	346.2
Wheat meal	50
Oats	50
Hypro soya	173.7
Full fat soybean	30
Whey powder	138.9
L-lysine HCl	3.6
DL-Methionine	1.9
L-Threonine	2.0
L-Tryptophan	0.2
L-Valine	0.9
Limestone	0.9
Dicalcium phosphate	1.7
Sodium chloride	1.2
Soya oil	18.8
Fish meal	75
Vitamin and mineral premix ^2^	5
Analysed nutrient composition, %	
Digestible energy, MJ/Kg	15.4
Crude Protein	22.25
Dry Matter	90.0
Calcium	0.87
Phosphorus	0.60
Phytate phosphorus	0.11
Ash	5.1
Fat	5.2
Neutral detergent fibre	8.8

^1^ Starter diets were created without or with (0 or 2500 FTU/kg) Quantum Blue 5G phytase (AB Vista, Marlborough, UK) with analysed phytase activities of 159 and 2580 FTU/kg, respectively; ^2^ Supplied per kg of diet: manganese, 62 mg; zinc, 99.9 mg; iron (ferrous sulphate), 200 mg; copper, 160 mg; iodine, 2.17 mg; selenium, 0.30 mg; retinol (vitamin A), 12.5 mg; cholecalciferol (vitamin D_3_), 2.0 mg; tocopherol (vitamin E), 200 mg; thiamine (vitamin B_1_), 4.2 mg; riboflavin (vitamin B_2_), 5.6 mg; pyridoxine (vitamin B_6_), 5.0 mg; cobalamin (vitamin B_12_), 50.0 mg; hetra (vitamin K), 4.4 mg; nicotinic acid, 40 mg; pantothenic acid, 19.99 mg; folic acid, 1.0 mg; and biotin 150 mg. choline chloride, 250 mg.

**Table 2:** Effect of creep feed and phytase on weaning pig performance ^1^.

		BW, kg			ADG, kg/d			ADFI, kg/d			FCR, kg:kg		
Creep Feed	Phytase, FTU/kg	d0	d7	d21	d0–7	d7–21	d0-21	d0–7	d7–21	d0–21	d0–7	d7–21	d0–21
No		6.6	7.6	14.1	0.14 ^b^	0.47	0.36	0.18	0.53	0.41	1.35 ^a^	1.13	1.15
Yes		7.1	8.7	15.5	0.23 ^a^	0.49	0.40	0.22	0.56	0.45	0.99 ^b^	1.17	1.13
	0	6.8	8.1	14.5	0.18	0.46	0.36	0.20	0.54	0.43	1.23	1.19	1.18 ^a^
	2000	6.8	8.2	15.1	0.19	0.49	0.39	0.20	0.55	0.43	1.11	1.11	1.10 ^b^
SEM		0.29	0.37	0.58	0.020	0.023	0.020	0.020	0.026	0.022	0.074	0.027	0.025
*p*-value													
Creep feed		0.255	0.057	0.127	0.011	0.611	0.171	0.164	0.341	0.248	0.005	0.323	0.640
Phytase		0.822	0.713	0.406	0.620	0.290	0.316	0.963	0.845	0.868	0.317	0.082	0.053
Creep feed × phytase		0.797	0.853	0.996	0.962	0.839	0.860	0.727	0.594	0.755	0.859	0.560	0.681

^1^ Least squares means (LSM) represent the main effects of eight replicate pens per treatment group (four pigs per pen); ^a,b^ LSM in each treatment that do not share a common superscript differ significantly (*p* ≤ 0.05); BW = body weight; ADG = average daily gain; ADFI = average daily feed intake; FCR = ADFI/ADG.

**Table 3:** Real-time stomach pH of piglets in response to creep feed during suckling and at weaning.

	7 Days Pre-Weaning			Day of Weaning		
Creep	Min	Max	Average ^1^	Min	Max	Average ^2^
No	3.00	5.25	4.55 ^a^	2.26	5.27	3.70 ^a^
Yes	2.01	4.21	3.24 ^b^	1.68	4.79	3.08 ^b^
SEM			0.087			0.124
*p*-value			<0.001			0.003

^1^ Least squares means (LSM) represent the average response of eight pigs; ^2^ LSM represent the average response of seven pigs; ^a,b^ LSM within the same column that do not share a common superscript differ significantly (*p* ≤ 0.01).

**Table 4:** Real-time stomach pH of piglets in response to phytase supplementation post-weaning.

	Day 7 Post-Weaning			Day 21 Post-Weaning		
Phytase, FTU/kg	Min	Max	Average ^1^	Min	Max	Average ^2^
0	1.99	5.20	3.27 ^a^	1.47	4.73	2.58 ^a^
2000	1.22	4.29	2.62 ^b^	1.27	3.76	2.15 ^b^
SEM			0.111			0.082
*p*-value			<0.001			0.005

^1^ Least squares means (LSM) represent the average response of six (0 FTU/kg phytase) and eight (2000 FTU/kg phytase) pigs; ^2^ LSM represent the average response of eight pigs; ^a,b^ LSM within the same column that do not share a common superscript differ significantly (*p* ≤ 0.01).

**Table 5:** Plasma *myo*-inositol concentrations and cortisol concentrations in weaning pigs.

		*Myo*-inositol (µM)			Cortisol (ng/mL)		
Creep	Phytase, FTU/kg	d0	d7	d21	d0	d7	d21
No		94.0 ^b^	44.3	31.3	87.1	32.0	10.3
Yes		112.8 ^a^	39.9	33.2	91.9	20.3	10.4
	0	-	39.3	21.7 ^b^	-	32.3	10.7
	2000	-	44.9	42.7 ^a^	-	20.0	10.0
SEM		6.07	3.28	3.07	16.24	6.95	1.45
*p*-value							
Creep feed		0.037	0.350	0.657	0.835	0.245	0.951
Phytase		-	0.243	<0.001	-	0.224	0.719
Creep feed × phytase		-	0.245	0.271	-	0.730	0.810

Least squares means (LSM) represent the main effects of eight pigs per treatment category; ^a,b^ LSM in each treatment that do not share a common superscript differ significantly (*p* ≤ 0.05).

## Data Availability

The dataset supporting this study is present within the article.
